# Microbial Delivery Vehicles for Allergens and Allergen-Derived Peptides in Immunotherapy of Allergic Diseases

**DOI:** 10.3389/fmicb.2018.01449

**Published:** 2018-07-02

**Authors:** Abida Zahirović, Mojca Lunder

**Affiliations:** Faculty of Pharmacy, University of Ljubljana, Ljubljana, Slovenia

**Keywords:** allergy immunotherapy, bacteriophage, delivery vehicle, lactic acid bacteria, S-layers, virus-like particle, viral surface protein

## Abstract

Allergen-specific immunotherapy represents the only available curative approach to allergic diseases. The treatment has proven effective, but it requires repetitive administrations of allergen extracts over 3–5 years and is often associated with adverse events. This implies the need for novel therapeutic strategies with reduced side effects and decreased treatment time, which would improve patients’ compliance. Development of vaccines that are molecularly well defined and have improved safety profile in comparison to whole allergen extracts represents a promising approach. Molecular allergy vaccines are based on major allergen proteins or allergen-derived peptides. Often, such vaccines are associated with lower immunogenicity and stability and therefore require an appropriate delivery vehicle. In this respect, viruses, bacteria, and their protein components have been intensively studied for their adjuvant capacity. This article provides an overview of the microbial delivery vehicles that have been tested for use in allergy immunotherapy. We review *in vitro* and *in vivo* data on the immunomodulatory capacity of different microbial vehicles for allergens and allergen-derived peptides and evaluate their potential in development of allergy vaccines. We also discuss relevant aspects and challenges concerning the use of microbes and their components in immunotherapy of allergic diseases.

## Introduction

Allergen-specific immunotherapy is based on the repeated administration of increasing doses of allergen extracts over 3–5 years (Akdis and Akdis, 2014). Although this conventional immunotherapy regimen has proven effective, several important weaknesses such as the high percentage of undesired IgE-mediated adverse effects and long treatment duration are forcing the development of novel immunotherapeutic approaches and preparations (Larsen et al., 2016). One possible approach relies on design of molecular allergy vaccines which include individual allergen proteins (recombinant allergens), allergen-derived peptides carrying relevant epitopes or epitope-mimicking peptides (mimotopes) (Szalai et al., 2008; Marth et al., 2014; Tscheppe and Breiteneder, 2017; Valenta et al., 2017). Allergen-derived peptides are designed to contain antigenic determinants of major allergens (epitopes) that are capable of activating the appropriate cellular and humoral responses while avoiding possible allergenic and/or reactogenic responses induced by whole allergens and allergen extracts. T cell epitope peptides are longer synthetic peptide sequences derived from primary allergen structure (Moldaver and Larché, 2011). B cell epitope peptides and mimotopes are short peptides that include or mimic three-dimensional IgE binding sites of allergens, respectively.

Recombinant allergens and particularly allergen-derived peptides are generally inadequately immunogenic and their immunogenicity is usually enhanced with application of an adjuvant (Siskind et al., 1966; Valenta et al., 1999). However, traditional agents and preparations with adjuvant properties are badly tolerated, and only a couple of them are appropriate for human use (Petrovsky, 2015). Aluminum hydroxide is the most widely used adjuvant in allergy immunotherapy with excellent safety record but has some limitations, particularly with regard to its profound T_H_2-biasing effects (Hogenesch, 2012). Therefore, current research aims to develop new, potent and effective delivery vehicles for allergens or allergen-derived peptides that are able to induce tolerance and analogous to CpG motifs exhibit T_H_1-immunostimulating properties (Johansen et al., 2005). An ideal delivery vehicle should possess the following characteristics: (1) provide targeted delivery and efficient presentation of vaccine components to the specific immune cells in a manner that would induce appropriate immune response (Moingeon et al., 2002; Souza et al., 2005); (2) exhibit low intrinsic immunogenicity to allow readministration in order to boost relevant specific immune response (Chesné et al., 2016); (3) sustain the vaccine release over an extended period of time; (4) protect vaccine components from degradation; and (5) allow large-scale production at low cost (Souza et al., 2005; Jafari and Abediankenari, 2016).

Over the recent years, viruses and bacteria have been intensively studied for their potential as delivery vehicles in allergy vaccines. Here, we discuss different aspects of microbial delivery vehicles of allergens and allergen-derived peptides employed in allergy immunotherapy in the attempt to develop formulations with improved immunogenicity and stability as well as the ability to target specific cells.

## Whole Viral and Bacteriophage Particles as Delivery Vehicles

**FIGURE 1:**
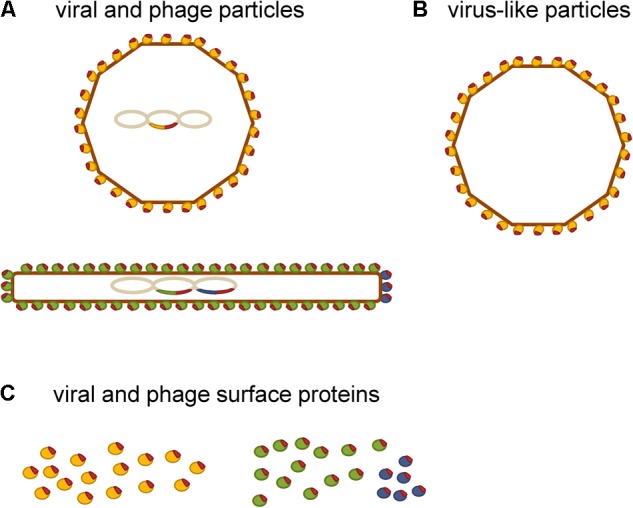
Schematic representation of viral delivery vehicles that have been tested for use in allergy immunotherapy. **(A)** Viral and phage delivery vehicles displaying allergen-derived peptides (red) on their surface. **(B)** Virus-like particles as delivery vehicles of allergens or allergen-derived peptides (red) that are incorporated into the virus-like particle either by chemical coupling or by recombinant expression as fusion proteins. **(C)** Single viral (yellow) and phage surface protein carriers (green and blue) of allergen-derived peptides.

Despite their inherent ability to induce humoral and cellular immune responses, a major obstacle in using eukaryotic viruses as delivery vehicles in humans is their potential pathogenicity and oncogenic integration into the genome of the host cells (Souza et al., 2005; Bakhshinejad and Sadeghizadeh, 2014; Jafari and Abediankenari, 2016). The presence of pre-existing immunity to the viral vector, which causes fast viral clearance from the body and thereby reduces the dose of the vectored antigen even before it is able to elicit an immune response, is another limitation to their general use (Souza et al., 2005; Saxena et al., 2013). These disadvantages have stimulated the search for novel more adequate vaccine delivery vehicles among non-eukaryotic viruses. **Figure 1** shows a schematic representation of different types of viral delivery vehicles that have been tested for use in allergy immunotherapy.

Bacteriophages (or phages for short) are viruses that infect bacteria. They are composed of DNA or RNA genome encapsulated inside a protein shield called capsid. In contrast to eukaryotic viruses, phages propagate in a prokaryotic host, and therefore appear as an attractive alternative for use in humans (Bakhshinejad and Sadeghizadeh, 2014; Jafari and Abediankenari, 2016). They act as inert particulate antigens, which are taken up and processed by antigen-presenting cells, and thus induce specific immune response by targeting delivery to these cells (Gao et al., 2010). The studies demonstrated that phages can induce both humoral and cellular immune responses without the use of an adjuvant (Adhya et al., 2014; Clark and March, 2014; Jafari and Abediankenari, 2016). Protection of the displayed peptides from a variety of harsh environmental conditions by phage particle provides extended degradation time and makes phages appropriate vectors for oral and mucosal applications (Delmastro et al., 1997; Jensen-Jarolim et al., 1998; Jepson and March, 2004). Combination of the feasibility of large-scale, cost-effective production and ease of modification makes them appealing for the industrial development of phage-based vaccines.

Phage-based vaccines are usually developed by recombinant fusion of the antigen to one of the virion surface proteins, of which the pIII and pVIII of M13 phage are used most frequently (**Figure 1A**). Such phage virions have the antigenic sequence included in their genome, which allows steady production of the vaccine construct through bacterial amplification (Aghebati-Maleki et al., 2016). Alternatively, the antigenic sequence may be conjugated to the phage surface via artificial linkers (van Houten et al., 2010). This allows a broader range of antigens to be displayed. However, the vaccine construct must be prepared by synthesis for each batch. The intensity of immune response depends on characteristics of the displayed peptide and the method of its display. For example, pIII fusions usually exhibit lower immunogenicity than pVIII fusions (Jensen-Jarolim et al., 1998; Scholl et al., 2002). The reason for this probably lies in the copy number difference. There are 2,700 copies of the major coat protein (pVIII) and only five copies of the minor coat protein (pIII) present on the surface of M13 filamentous phage (**Figure 1A**). Namely, an antigen displayed in high copy on the surface of an individual phage virion is more effective in eliciting an immune response than the same antigen displayed in low copy number (Rakonjac et al., 2011). In general, phages possess comparable immunogenicity to that of the traditional carrier proteins such as bovine serum albumin or keyhole limpet hemocyanin and essentially have a small number of their own B cell epitopes to deflect antibody response away from the molecule they display (Luzar et al., 2016b). Indeed, it has been shown that filamentous phage carriers elicit antibody response that is more focused against displayed peptides compared to the traditional carrier protein OVA (van Houten et al., 2006). Although still in early stages of development, phage-based vaccines have been used to induce protection against infectious diseases and cancers in preclinical studies and have also been tested in phase I/II clinical studies (Roehnisch et al., 2014). Both icosahedral phages (such as lambda, T7, T4) and filamentous phages (such as fd, F1, M13) have been used for production of these vaccines (Jafari and Abediankenari, 2016).

In allergy immunotherapy, filamentous phage M13 has been most commonly employed for delivery of allergen-derived peptides (Chen and Dreskin, 2017). In the study performed by Luzar et al. (2016a), the filamentous phage particles displaying mimotopes of major cat allergen Fel d 1 (approximately 150 copies fused to major coat protein pVIII) were constructed and evaluated for their potential as vehicles for a cat allergy vaccine. Even though the mimotopes recognized IgE from sera of cat-allergic patients they did not activate the basophils of these patients. The phage carrier on the other hand caused non-specific stimulation of basophils probably triggering receptors of innate immunity such as Toll-like receptors, which are known to be present on basophil surface (Suurmond et al., 2014). Importantly, phage-displayed mimotopes were able to induce preferentially T_H_1 directed response (increased IFN-γ production) in cultures of PBMCs from allergic patients.

The ability of phage-displayed mimotopes to induce antibodies specific for the whole allergen has been demonstrated in animal studies. In a mouse model, intragastric as well as intranasal administration of phages displaying mimotopes of major birch pollen allergen Bet v 1 (approximately 2,700 copies fused to major coat protein pVIII) induced Bet v 1-specific IgG response (Jensen-Jarolim et al., 1998; Scholl et al., 2002). Unfortunately, this IgG failed to induce immune tolerance to Bet v 1 in skin reactivity test emphasizing the importance of peptides’ ability to adequately mimic the IgE epitopes on allergen (Knittelfelder et al., 2009). Since epitope specificity of an induced IgG antibody can be decisive for the success of therapy, particular attention must be paid to this issue during the development of peptide-based vaccines.

The studies suggest a possible future for filamentous phages as delivery vehicles in the therapy of allergic reactions. Nevertheless, several important drawbacks currently limit their application in clinical practice. Although the phage therapy has proven safe in healthy human volunteers (Bruttin and Brussow, 2005), some phages have the potential to release endotoxin from lysed Gram-negative bacteria (Young, 1992). This concern can be avoided with the application of non-lytic filamentous phages (e.g., M13 phage). However, when administered via oral route, these phages may transfer virulence factors or genes that confer antibiotic resistance to F-pili positive intestinal microbiota and thereby generate new unwanted traits (Bazan et al., 2012; Colavecchio et al., 2017). Additionally, long-term treatments with phages or phage exposure itself can induce an antibody response against phages, which can decrease their titer and reduce the effectiveness of therapy (Clark and March, 2014). Therefore, substantial evidence acquired in clinical trials, particularly regarding phage safety and effectiveness in subjects, who are positive for anti-phage antibodies, is still missing.

## Self-Assembling Virus-Like Particles as Delivery Vehicles

Virus-like particles are composed of one or several viral structural proteins that have the ability to self-assemble during recombinant expression (Fuenmayor et al., 2017). They resemble and mimic the structure of actual viruses (Zeltins, 2013). A key advantage of VLPs is the lack of viral genomic material, which enhances safety during both manufacture and administration (Klimek et al., 2014). VLPs are composed of many subunits of one or more viral capsid proteins, which can be modified to display short peptide sequences in high-density at their surface either by genetic engineering or by chemical coupling, as schematically represented in **Figure 1B** (Brown et al., 2009; Schmitz et al., 2009). The findings in mouse models showed that an antigen presented to the immune cells in a highly ordered repetitive fashion is capable of eliciting strong antibody response even in the absence of adjuvant, while the same antigen presented as a monomer appears to be non-immunogenic (Feldmann and Easten, 1971; Marth et al., 2013). Examples of such natural repetitive immunogenic structures are surfaces of viruses and bacteria. There is also epidemiologic evidence that the repetitiveness of antigen correlates with its immunogenicity for B cells in human subjects (Jegerlehner et al., 2002a). This was essentially the rationale for the use of VLPs as carriers of allergens and allergen-derived peptides.

First, Jegerlehner et al. (2002b) showed that the antigens displayed on VLPs derived from the 180 coat protein subunits of the bacteriophage Qβ (Qβ-VLPs) are highly immunogenic in mice. A strong IgG2-dominated antibody response was induced by Qβ-VLPs because of the presence of bacterial host RNA, which is a ligand of Toll-like receptors. It was encapsulated into the VLPs during self-assembly process (Forsbach et al., 2007). Similarly, Qβ-VLPs have been shown to greatly enhance the immunogenicity of major cat allergen Fel d 1 chemically coupled to their surface (Schmitz et al., 2009).

In the first human use of VLP-based vaccines, Kündig et al. (2006) generated a construct composed of a peptide sequence from major house dust mite allergen Der p 1 chemically coupled to bacteriophage Qβ coat protein and evaluated its safety and immunogenicity in phase I clinical trial. Twenty-four healthy volunteers were vaccinated by two different routes (subcutaneous and intramuscular) with two different doses (10 μg and 50 μg of total protein) without the use of an adjuvant. The treatment was well tolerated. All immunized subjects developed a significant antibody response to both Der p 1 and bacteriophage Qβ coat protein, even after single injection demonstrating that allergen coupled to highly repetitive VLPs is an efficient approach for rapid induction of high titers of antibodies in human subjects (Kündig et al., 2006). The response depended on the administered dose, while the immunization route had only small influence. These findings were extended in phase I/IIa clinical trial, in which Senti et al. (2009) investigated the safety, tolerability and clinical effectiveness of treatment composed of house dust mite extract and deoxynucleotides with CpG motifs packaged into Qβ-VLPs. CpG motifs are known ligands of Toll-like receptor 9 with T_H_1-immunostimulating properties (Johansen et al., 2005). Twenty-one dust mite allergic patients were enrolled in an open monocentric study. The results showed high level of safety and good tolerability. The treatment led to increased allergen-specific IgG and reduced skin reactivity to house dust mite extract. Almost complete tolerance to the allergen in conjunctival provocation testing and a significant reduction of rhinitis and allergic asthma symptoms were observed. After 10 weeks of treatment, patients were almost without symptoms. This alleviation lasted for at least 38 weeks after the treatment (Senti et al., 2009).

Virus-like particles obtained from adeno-associated viruses (AAVLPs) are composed of 60 copies of the VP3 capsid protein, which can be genetically modified to display short peptide sequences. Manzano-Szalai et al. (2014) assessed on a mouse model the immunogenicity and safety of such AAVLPs displaying a B-cell epitope peptide of food allergen OVA. The results showed that the titers of IgG1 specific for OVA in mice immunized with AAVLP-OVA were comparable to those induced by native OVA. However, native OVA elicited high levels of IgE, whereas OVA displayed on AAVLPs produced background IgE values only. Accordingly, OVA-immunized mice, but not mice immunized AAVLP-OVA, developed an anaphylactic reaction upon intravenous allergen challenge, which manifested as a significant drop in body temperature (Manzano-Szalai et al., 2014).

## Viral and Phage Surface Proteins as Delivery Vehicles

Different viral and phage surface proteins have also been tested for delivery of allergen-derived peptides (**Figure 1C**). In a number of preclinical studies, they showed excellent immunomodulatory capacity (Focke et al., 2001, 2010; Niespodziana et al., 2011; Valenta et al., 2016) and proved to be suitable for clinical trials (Zieglmayer et al., 2016).

### VP1 Surface Protein From Human Rhinovirus

A study by Edlmayr et al. (2009) reported the construction of a recombinant vaccine for grass pollen allergy using surface protein VP1 from rhinovirus, which plays a major role in viral infection of respiratory cells. Recombinant fusion proteins composed of VP1 and a B cell epitope peptide derived from the major grass pollen allergen Phl p 1 were not recognized by patients’ IgE and showed no allergenic activity in basophil activation test. Immunization of mice and rabbits with the fusion proteins resulted in the production of IgG that cross-reacted with group 1 grass pollen allergens. The induced antibodies were able to block recognition of native Phl p 1 by patients’ IgE and Phl p 1-induced activation of basophils.

### Hemagglutinin A Surface Protein From Influenza A Virus

Hemagglutinin A is a dominant glycoprotein on the envelope of influenza virus and a key antigen in the host response to virus infection. In a study conducted by Mrkić et al. (2016), the immunomodulatory potential of recombinant chimeric protein composed of the major allergen of house dust mite, Der p 2, and hemagglutinin A was tested in a mouse model. Intranasal pretreatment of mice with Der p 2/hemagglutinin A fusion, prior to sensitization with the allergen, significantly decreased IgE levels and markedly increased allergen-specific IgG and IgA levels in sera. Moreover, enhanced proliferation of CD4+CD25+ regulatory T cells was detected in mouse spleens after pretreatment with fusion molecule but not with the native allergen. This indicates that the carrier beneficially affects the immunomodulating properties of the vaccine.

### PreS Surface Protein From Hepatitis B Virus

The PreS domain is a part of a large surface protein, which forms the hepatitis B virus envelope along with middle and small surface proteins. It showed good immunogenicity and safety during clinical use as a hepatitis B vaccine (Ilaria et al., 2016). Recently, several recombinant fusion proteins composed of PreS and B cell epitopes derived from allergens Fel d 1 (Niespodziana et al., 2011), Der p 23 (Banerjee et al., 2014), and Bet v 1 (Marth et al., 2013) have been constructed as vaccine candidates. The recombinant fusion proteins showed no relevant IgE reactivity and strongly reduced allergenic activity. Immunization of animals resulted in the production of allergen-specific IgG that inhibited the binding of allergic patients’ IgE to the native allergen as well as allergen-induced activation of basophils to a similar extent or better as did IgG elicited by vaccination with native allergen (Marth et al., 2013). This indicated that some of these fusion proteins have the ability to focus IgG response against the major IgE-reactive sites on allergen better than the allergen itself. In PBMCs from allergic patients, lower T cell proliferation and lower levels of T_H_2 cytokine IL-5 were observed compared to the effect of native allergens. This was additionally associated with the secretion of the higher levels of the tolerogenic cytokine IL-10 and the T_H_1-specific cytokine IFN-γ (Banerjee et al., 2014).

In the study conducted by Focke-Tejkl et al. (2015), four fusion proteins composed of B-cell epitope peptides from the major timothy grass pollen allergens (Phl p 1, Phl p 2, Phl p 5, and Phl p 6) and the PreS were generated and evaluated as components of the vaccine termed BM32. The BM32 vaccine, whose allergenic activity was almost completely eliminated, showed significantly reduced T-cell proliferation and decrease in production of proinflammatory cytokines in patients’ PBMCs compared to grass pollen allergens. The vaccine was capable of inducing specific IgG antibodies directed toward native allergens in mice. Moreover, induced IgG were able to inhibit the binding of patients’ IgE to all four major grass pollen allergens as well as inhibit the activation of basophils by the allergens. BM32 is at present farther advanced B-cell epitope-based vaccine. In the most recent multicentered double-blind, placebo-controlled phase 2b clinical trial, BM32 was well tolerated and efficiently relieved symptoms of allergic rhinitis in patients (Zieglmayer et al., 2016; Niederberger et al., 2018).

### PIII Surface Protein From M13 Phage

Multivalent display of antigenic epitopes provides high immunogenicity to the therapeutic constructs and is desirable in most cases. However, this may present a limitation in the context of mimotope immunotherapy. Since mimotopes imitate the natural IgE epitopes and normally bind IgE, they might cross-link IgE on effector cells if displayed in high density on a carrier. Therefore, the application of monovalent carriers of allergen mimotopes may be more favorable. We tested the minor coat protein pIII from M13 phage as a delivery vehicle for mimotopes of major bee venom allergen Api m 1. PIII-fused mimotopes were recognized specifically by patients’ IgEs, thus demonstrating that they imitate the natural IgE epitope; however, they caused no basophil degranulation in corresponding patients. This confirmed the absence of allergenic activity and demonstrated that the mimotopes bound to a monovalent carrier such as the minor coat protein pIII are not able to cross-link IgE on basophils. In addition, pIII-fused mimotopes exhibited immunomodulatory effects by eliciting secretion of T_H_1 cytokine IFN-γ in PBMCs from bee venom-allergic patients, as opposed to bee venom and Api m 1, indicating a shift from T_H_2 toward T_H_1 immune response. These results suggest that the minor coat protein pIII might be suitable as a delivery vehicle for mimotopes obtained from phage display libraries. By using single coat proteins the problems associated with the application of whole phages in humans such as their potential to transfer antibiotic resistance to F-pili positive microbiota can also be avoided. Furthermore, the mimotopes fused to pIII preserve the conformation they had when displayed on phage. Hence, we can circumvent the problems with the loss of mimicry potential which was observed in the case of chemical coupling of mimotopes to certain traditional protein carriers such as keyhole limpet hemocyanin (May et al., 2003).

## Live Bacteria as Delivery Vehicles

**FIGURE 2:**
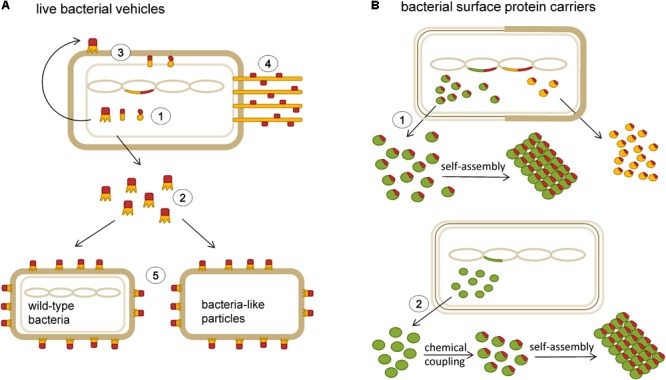
Schematic representation of bacterial delivery vehicles that have been tested for use in allergy immunotherapy. **(A)** Live bacterial vehicles with various modes of delivery of allergens or allergen-derived peptides (red): intracellular expression (1), secretion (2), membrane- and cell wall display (3), pilus-mediated display (4); heterologous display (5). **(B)** Bacterial surface protein carriers; S-layer proteins (green) and albumin binding protein (yellow): recombinant expression of carrier proteins with allergen-derived peptides (1), isolation of surface proteins and chemical coupling to allergens or allergen-derived peptides (2).

Owing to their immunomodulatory properties, a number of probiotic strains have shown beneficial effects in the treatment of allergic diseases (Repa et al., 2003; Karimi et al., 2009; Costa et al., 2014; Ai et al., 2015b; Yepes-Nunez et al., 2016). In light of this, recombinant LAB engineered to produce and/or deliver allergens or allergen-derived peptides to mucosal surfaces to induce tolerance have emerged. **Figure 2** shows a schematic representation of different types of bacterial delivery vehicles that have been tested for use in allergy immunotherapy. Allergen vaccination via mucosal route is a desirable alternative to subcutaneous injections. It is not only easier but also increases the effectiveness against allergens that enter the body through mucosal surfaces (Wyszyńska et al., 2015). Some LAB strains have adjuvant properties and can enhance the immune response to the carried antigen (Schabussova and Wiedermann, 2008). They are especially suited for human use because of their “GRAS” status (Berlec et al., 2012; Trombert, 2015). Furthermore, LAB ability to withstand the passage through the gastrointestinal tract makes them an ideal oral delivery vehicle (Hynonen and Palva, 2013). Gut colonization by live strains allows the reduction of the number of vaccine doses required and also simplifies the immunization procedure to a great extent. Recombinant LAB serve as production hosts and as protective coatings at the same time. This can result in lower costs, as there is less need for purification of allergen protein and development of formulation. Additionally, lyophilization of LAB increases their stability at room temperature (Berlec et al., 2012). All of these characteristics, particularly, the immunomodulating and adjuvant effects as well as high safety profile, make LAB an attractive delivery vehicle for the construction of allergy vaccines. Live bacterial vehicles with intracellular, cell wall or membrane based display of antigens are schematically represented in **Figure 2A**.

### Mucosal Delivery of Allergen-Expressing LAB in Food Allergy

In the context of food allergy, oral pretreatment of mice with *L. lactis* strains secreting β-lactoglobulin, the major cow’s milk allergen, induced a shift toward T_H_1 immune response and reduced the IgE levels. The best results were obtained with the strains that produced the highest amounts of β-lactoglobulin (Adel-Patient et al., 2005). In another study, similar effects on mice were obtained with *Lb. casei* expressing the same allergen (Hazebrouck et al., 2006).

Oral tolerance is an unresponsiveness of gut immune system to innocuous food antigens ingested by oral route. This process is regulated by multiple mechanisms, in which the dose of the antigen has an important role. High doses of antigen induce deletion or anergy of the T cells recognizing the antigen, whereas low doses induce antigen-specific regulatory T cells, which promote active suppression through secretion of tolerogenic cytokines (such as IL-10 and transforming growth factor β). Induction of regulatory T cells is a major goal for immunotherapy of allergic diseases, and it can be achieved by exposing the mucosal immune system to low doses of antigen (Mowat, 2003). Accordingly, the application of *L. lactis* secreting egg ovalbumin (LL-OVA) to transgenic mice with expressed OVA-specific T cell receptor on CD4+ T cells caused a reduction in delayed-type hypersensitivity responses to OVA. This reduction was mediated by induction of CD4+CD25+ regulatory T cells that secreted transforming growth factor β. Restimulation of splenocytes and gut-associated lymph node tissue from these mice with OVA caused reduced IFN-γ and increased IL-10 production. The effect was achieved with substantially lower doses of OVA (up to 10 μg), secreted in the gut upon repeated oral administration of LL-OVA, compared to that typically used for successful tolerance induction (5 mg). Interestingly, control *L. lactis* carrying the empty vector also suppressed OVA-specific delayed-type hypersensitivity responses albeit to a lesser extent than LL-OVA. Contrary to the control, induction of regulatory T cells was detected only in mice fed with LL-OVA (Huibregtse et al., 2007). The mechanism by which wild-type *L. lactis* suppressed OVA-specific delayed-type hypersensitivity response remains to be explored. Whether administration of *L. lactis* simply mixed with low doses of soluble OVA also induces oral tolerance is worth exploring as it can overcome the need to use recombinant bacteria.

### Mucosal Delivery of Allergen-Expressing LAB in Respiratory Allergies

In studies involving inhalational allergies, oral administration of *Lb. plantarum* expressing major dust mite allergen Der p 1 and *Lb. acidophilus* expressing another major dust mite allergen Der p 5 reduced hyperreactivity and inflammation of the airways induced by allergen and decreased the production of specific IgE (Charng et al., 2006; Rigaux et al., 2009). Also, in a murine model of cedar pollinosis *Lb. plantarum* secreting major Japanese cedar pollen allergen Cry j 1 was able to suppress nasal clinical symptoms and allergen-specific IgE response upon oral administrations (Ohkouchi et al., 2012). Similarly, recombinant *L. lactis* and *Lb. plantarum* that produce inhalational allergen Bet v 1 were evaluated for their immunomodulating potential in a murine model of birch pollen allergy. A prophylactic intranasal immunization of mice, caused an increased production of allergen-specific IgA and induced a shift toward T_H_1-specific immune response (Daniel et al., 2006). Different routes of application were compared and intranasal application seemed more effective than the intragastric route. Moreover, *Lb. plantarum* was more effective carrier compared to *L. lactis*. This was attributed to lower production of Bet v 1 and shorter intestinal transit time of *L. lactis* (Daniel et al., 2007). Therefore, the intrinsic immunomodulating capacities of the strain, gut persistence, or both, are important contributing factors. Additionally, cellular location of produced allergen showed influence on the treatment efficacy. The Bet v 1 secreting strain of *Lb. plantarum*, particularly after intranasal pretreatment, led to a stronger reduction of allergen-specific IgE and higher secretion of T_H_1-dependent IgG2a compared to the *Lb. plantarum* that produced the allergen intracellularly (Daniel et al., 2007).

Recently, pre-, peri- and/or postnatal periods have been proposed as a critical short-time interval when many factors influence the onset and course of allergic diseases. Several clinical studies have found an association between a reduced number of lactobacilli or bifidobacteria in the early intestinal microbiota of atopic children and the occurrence of allergic diseases later in life (Marschan et al., 2008). Thus, intervening at an early developmental stage seems reasonable targeted preventive strategy to modulate immune responses in a long term. Indeed, this was confirmed in a study conducted by Schwarzer et al. (2011), in which germ-free mice were colonized with the *Lb. plantarum* producing Bet v 1 and did not develop birch pollen allergy later on. The effect was associated with an increased immunoregulatory responses and a shift to a non-allergic T_H_1 phenotype. Notably, in this study the wild-type *Lb. plantarum* itself had no suppressive effects on the allergic immune response. The effects depended on the expression of the specific allergen.

Besides allergens, allergen-derived T cell epitope peptides displayed on LAB were evaluated for active vaccination and induction of tolerance in allergy. Mucosal immunization of mice with peptide from the house dust mite followed by treatment with recombinant *Lb. plantarum* expressing an immunodominant T-cell epitope of major dust mite allergen Der p 1 inhibited production of both IFN-γ and IL-5. The effect on IFN-γ was shown to be a non-specific effect of *Lb. plantarum*, while the effect on IL-5 production was observed only when the *Lb. plantarum* expressing Der p 1-derived peptide was used for treatment (Kruisselbrink et al., 2001). Similarly, *L. lactis* was engineered to express peptides containing major T cell epitopes of another major dust mite allergen Der p 2 and their protective effects were evaluated in Der p 2-sensitized BALB/c mice model. Mucosal delivery of these strains reduced specific IgE levels and decreased lung inflammatory responses caused by Der p 2. The study showed an increase of specific IgG2a in serum and a proliferation of regulatory T cells in mesenteric lymph nodes in association with the protective responses (Ai et al., 2015a).

*L. lactis* produces a cell envelope structural component known as a polysaccharide pellicle, which might restrict accessibility of the heterologous peptide (Chapot-Chartier et al., 2010). This can be circumvented by displaying foreign peptides on the tip of a pilus to expose the peptides at a distance from the cell envelope (see **Figure 2A** for schematic representation) (Quigley et al., 2009). The pilus structure is also highly immunogenic. In the proof-of-principle study, the peptide from the major egg allergen ovomucoid (Ova324–339) was inserted into three different loop regions of the monomeric pilus backbone protein from group A *Streptococcus pyogenes* serotype M1 (PilM1) and expressed in high copy number as a part of pili on the surface of *L. lactis* (LL-PilM1-Ova). Intranasal immunization of mice with LL-PilM1-Ova generated measurable Ova-specific systemic and mucosal responses (IgA and IgG). Notably, Ova-specific IgG or IgA were not detected in serum or mucosal sites when synthetic Ova324–339 was mixed with LL-PilM1. This indicates that the adjuvant property of *L. lactis* alone is not sufficient to induce Ova-specific immunity and suggests that the physical integration of the peptide into the pilus structure is important, probably due to peptide stabilization and prevention of enzymatic degradation (Wagachchi et al., 2018). This method seems to be a promising strategy for display of allergen-derived peptides on the LAB and remains to be compared with other modes of delivery (e.g., intracellular, cell wall anchored).

Taken together, these studies have shown that recombinant LAB pose as efficacious live vehicles that elicit specific and protective immune responses against the allergens or allergen-derived peptides. However, none of these constructs have been tested in human studies thus far. Several disadvantages of the engineered recombinant LAB have prevented their wider use in therapy. In the case of *in vivo* production of therapeutic molecules, the precise dosage is difficult to control. Moreover, the fate of the bacteria in the intestine and pharmacokinetics are difficult to determine (Berlec et al., 2012). Importantly, the major hindrance has been the fear of release of the genetically modified organism into the environment. Even though this has been successfully tackled by the development of containment system for *L. lactis* (Steidler et al., 2003) regulatory authorities will probably prefer the use of killed bacteria or BLPs (Berlec et al., 2012). BLPs are a non-recombinant alternative to live bacteria. They are obtained by treatment with hot trichloroacetic acid, which causes depletion of surface lipoteichoic acids, proteins, and the cytoplasmic content. The remaining intact peptidoglycan layer retains the particle shape similar to that of live cells (van Roosmalen et al., 2006). Because of the lack of recombinant DNA the risk of uncontrolled spreading of genetically modified material into the environment is eliminated. Another interesting approach to avoid the use of recombinant LAB is based on the non-covalent heterologous surface display of fusion proteins on unmodified, wild-type LAB (Hu et al., 2011; Zadravec et al., 2015a,b). In both cases, foreign proteins are produced as fusions with cell-wall binding domains in the recombinant host and are subsequently mixed with either BLPs or unmodified, wild-type LAB (see **Figure 2A** for schematic representation). These platforms allow the simultaneous presentation of several antigens, which may be significant for the production of vaccine candidates composed of several important allergens. Although these non-recombinant display technologies have not yet been tested in allergy immunotherapy, they open up new possibilities for improvement of allergy vaccine formulations.

## Bacterial Surface Proteins as Delivery Vehicles

### S-layer Proteins

Bacterial surface S-layers are two-dimensional crystalline arrays of glycoprotein subunits that make up the outermost layer of many bacteria (**Figure 2B**). S-layers have been shown to possess strong adjuvant properties and represent excellent carrier candidates for immunotherapeutic vaccines (Raha et al., 2005; Sleytr et al., 2014). The general applicability of S-layers as vaccine carriers for treatment of type I allergy was tested using S-layer self-assembly products from *Lysinibacillus sphaericus* or *Thermoanaerobacter thermohydrosulfuricus* chemically conjugated with recombinant Bet v 1 (Jahn-Schmid et al., 1996). In a subsequent study by Jahn-Schmid et al. (1997), T cell lines derived from PBMCs of birch pollen-allergic patients were induced either using recombinant Bet v 1 alone or Bet v 1/S-layer conjugates. After re-stimulation with Bet v 1, T cell lines induced with conjugates showed substantial increase in IFN-γ production compared to T cell lines induced with allergen only. The presence of IFN-γ in the induction phase of T cell lines has been described to lead to a preferential development of T cell clones with a T_H_1-like phenotype. Indeed, most of the T cell clones derived from the Bet v 1-induced T cell lines (55%) exhibited a T_H_2-like pattern of cytokine production and majority (79%) of the T cell clones established with the Bet v 1/S-layer conjugates revealed T_H_1 pattern. In PBMC cultures stimulation with S-layer proteins and Bet v 1/S-layer conjugates but not recombinant Bet v 1 increased production of IL-12, an essential mediator of T_H_1 response. This indicates an adjuvant effect of S-layer mediated by IL-12 (Jahn-Schmid et al., 1997).

In the following years, recombinant fusion of the Bet v 1 to S-layer proteins successfully replaced the procedures of chemical coupling. For example, recombinant fusion of Bet v 1 with S-layer proteins, SbpA from *Bacillus sphaericus* and SbsC from *Bacillus stearothermophilus*, yielded two S-layer/allergen recombinant constructs, which showed strongly reduced capacity to bind IgE compared to free Bet v 1 and possessed the ability to induce allergen-specific T_H_0/T_H_1 and regulatory T cell immune responses (Breitwieser et al., 2002; Ilk et al., 2002; Gerstmayr et al., 2007). Initially, the S-layer/allergen fusion proteins were expressed in Gram-negative host *E. coli* and the associated endotoxin was subsequently removed by purification procedure, which is costly and time-consuming. In a recent study a Gram-positive, non-pathogenic bacteria with naturally high secretory capacity, *Bacillus subtilis*, was tested for expression of the endotoxin-free recombinant protein. The obtained fusion protein consisting of Bet v 1 and S-layer surface protein SbpA from *Lysinibacillus sphaericus* showed excellent recrystallization properties and immune reactivity (Ilk et al., 2011). Bacterial S-layers also proved to be applicable as carriers for the development of a peanut allergen-derived peptides. In a study by Anzengruber et al. (2017), a fusion protein of the S-layer protein SlpB from *Lb. buchneri* and the peptide AH3a42, containing immunodominant B-cell epitopes and one T cell epitope of major peanut allergen Ara h 2, was generated. The fusion protein SlpB-AH3a42 was recognized by IgE from 69% of the allergic patients and did not induce β-hexosaminidase release from sensitized rat basophil leukemia cells. However, IgG antibodies induced by immunization of rabbits with the SlpB-AH3a42 molecule weakly inhibited IgE-binding to the natural Ara h 2 (no more than 30% reduction observed with 20 patient sera) in comparison with the inhibition by anti-Ara h 2 rabbit IgG antibodies (48% reduction). These results indicate that more than one peptide, derived from allergen, would probably be needed to promote wider patient protection.

### Albumin Binding Protein

In the study by Ganglberger et al. (2001), the Bet v 1 mimotopes were expressed as fusion proteins with streptococcal albumin binding protein as a monovalent carrier and their antigenicity and allergenicity were examined. The fusion proteins were shown to selectively bind to anti-Bet v 1 human IgE thus demonstrating that the mimotopes fused to albumin binding protein resemble the genuine IgE epitopes. Even though they possess IgE binding structures, the recombinant mimotope-albumin binding protein constructs did not cause skin test reactivity in Bet v 1-allergic mice, indicating that mimotopes of IgE epitopes are safe for immunotherapy when presented in a monovalent form. Furthermore, upon vaccination of BALB/c mice, the constructs were able to induce Bet v 1-specific IgGs that inhibited recognition of Bet v 1 by patients’ IgE.

## Conclusion

Microbial delivery vehicles have been applied in allergy immunotherapy to enhance its efficacy, reduce side effects, and shorten the treatment. Several promising viral and bacterial carriers have been developed and tested. Regarding phage carriers, apart from regulatory constraints, the pre-existing immunity and possible transfer of antibiotic resistance prevent their broader application in spite of the exceptional stability, cost-effectiveness, and ease-of-production. In general, delivery vehicles that are not genetically modified and are not problematic from the regulatory point of view are gaining momentum in today’s research. From this perspective, non-recombinant alternatives to GRAS probiotic carriers displaying particularly good performance in mucosal and gastrointestinal delivery are receiving attention. However, none of the proposed whole bacterial or whole viral delivery vehicles have reached the clinical phase of investigation thus far.

Despite numerous applications which have been proposed and their proof-of-principle demonstrated on animal models, only two virus-derived carriers have entered human trials. VLPs (in combination with CpGs) showed suitable clinical tolerance and beneficial immunological and clinical effects. Based on their viral immunomodulatory properties, VLPs in general and CpGs as adjuvants were successfully used in the treatment of allergic rhinitis. However, large controlled studies are needed to collect more extensive clinical experience with this new technology. Hepatitis B virus PreS surface protein is another viral carrier that successfully underwent phase 2b clinical trial, in which it proved to be safe and effective as a vehicle for subcutaneous delivery of B cell epitope peptides in grass pollen allergy.

In the future, the expression of multiple allergens or allergen-derived peptides on a single carrier will probably be necessary to cover a larger repertoire of the epitopes and to elicit optimal anti-allergic immune responses. This strategy also opens possibilities for patient-specific immunotherapy. Knowledge of the epitopes characteristic for the individual allergic patient, together with technology for development of the appropriate carriers, would allow targeted, personalized therapy. Moreover, the combination of several functional molecules (e.g., delivery vehicle, adjuvant, and allergen; such as VLP/CpG/house dust mite allergens) might be required to maximize vaccine efficiency. Finally, a deeper understanding of cellular and humoral factors involved in immune responses will contribute to optimization of these delivery systems and their faster translation to clinical practice.

## Author Contributions

ML conceived of the presented idea. AZ reviewed the literature and collected the data. Both ML and AZ contributed to the final version of the manuscript.

## Conflict of Interest Statement

The authors declare that the research was conducted in the absence of any commercial or financial relationships that could be construed as a potential conflict of interest.

## Abbreviations

AAVLP: adeno-associated virus-like particles
BLPs: bacteria-like particles
CpG: cytosine-phosphate-guanine deoxynucleotides
GRAS: generally regarded as safe
IFN: interferon
Ig: immunoglobulin
IL: interleukin
LAB: lactic acid bacteria
LL-OVA: OVA-secreting *L. lactis*
OVA: ovalbumin
PBMC: peripheral blood mononuclear cell
Qβ-VLPs: bacteriophage Qβ derived VLPs
T_H_1: type 1 helper cells
VLP: virus-like particle
